# The genome sequence of common ivy, *Hedera helix *L., 1753

**DOI:** 10.12688/wellcomeopenres.19662.1

**Published:** 2023-07-26

**Authors:** Maarten J. M. Christenhusz, David Bell, Alex D. Twyford, Royal Botanic Gardens Kew Genome Acquisition Lab, Royal Botanic Garden Edinburgh Genome Acquisition Lab, Plant Genome Sizing collective, Darwin Tree of Life Barcoding collective, Wellcome Sanger Institute Tree of Life programme, Wellcome Sanger Institute Scientific Operations: DNA Pipelines collective, Tree of Life Core Informatics collective, Darwin Tree of Life Consortium

**Affiliations:** 1 Royal Botanic Gardens Kew, Richmond, England, UK; 2 Royal Botanic Garden Edinburgh, Edinburgh, Scotland, UK; 3 The University of Edinburgh, Edinburgh, Scotland, UK

**Keywords:** Hedera helix, common ivy, genome sequence, chromosomal, Apiales

## Abstract

We present a genome assembly from a specimen of *Hedera helix* (common ivy; Streptophyta; Magnoliopsida; Apiales; Araliaceae). The genome sequence is 1,199.4 megabases in span. Most of the assembly is scaffolded into 24 chromosomal pseudomolecules. The mitochondrial and plastid genomes have also been assembled and are 609.2 and 162.2 kilobases in length respectively.

## Species taxonomy

Eukaryota; Viridiplantae; Streptophyta; Embryophyta; Tracheophyta; Spermatophyta; Magnoliopsida; eudicotyledons; Gunneridae; Pentapetalae; asterids; campanulids; Apiales; Araliaceae; *Hedera*; *Hedera helix* (Linnaeus 1753) (NCBI:txid4052).

## Background

The common ivy, *Hedera helix L.,* is a vigorous self-clinging evergreen perennial vine that is one of the most familiar plant species in the British flora. Ivy is common across most of Britain, except parts of northern Scotland. It is much rarer in Ireland, but it is native to much of Europe from southern Scandinavia to Turkey. It forms dense ground cover, particularly in secondary woodland. Here, it acts as a keystone species, with the greenish-yellow flowers providing a rich source of nectar for insects in the autumn and the purplish-black berries being a source of food for birds in the spring ( Metcalfe, 2005). The common ivy, as well as other introduced ivy species, are widespread in and around gardens, where they are used as climbers to cover garden structures, particularly in shaded situations. Common ivy has widely escaped from gardens and is now frequently found as a non-native alien around the globe ( Biggerstaff & Beck, 2007), usually then known as English ivy. The species is also notable for its rich folklore, and is still widely used in wreaths and celebrated in Christmas songs.

The taxonomy of ivy is complex, with disagreement around the recognition of subspecific taxa and confusion around the European native status of some horticultural taxa. Here, we follow Stace (2010), with our specimen belonging to the widespread diploid *Hedera helix* (syn. *Hedera helix* subsp. *helix*) which has 2 *n* = 48, rather than the more westerly distributed tetraploid *Hedera hibernica* Poit. (syn. *H. helix* subsp. *hibernica* (Poit.) D.C.McClint.; 2 *n* = 96).

Here, we present the first high-quality ivy genome, which we anticipate being a valuable genomic resource for a range of future studies. These may include investigations into the biosynthetic pathway underlying the production of triterpenoid saponins, which has previously been studied in ivies using transcriptomic data ( Sun *et al.*, 2017). The species is also of interest for its developmental genetics ( Schäffner & Nagl, 1979). The species undergoes a dramatic transition in leaf shape, with juveniles producing five-lobed leaves, while adults produce radially symmetrical ovate leaves ( Metcalfe, 2005). Finally, ivy is the host plant of the parasitic ivy broomrape ( *Orobanche hederae Duby*), and comparative genomic analyses could be used to investigate interactions in this obligate parasite-host system ( Twyford, 2018).

## Genome sequence report

**Figure 1.**
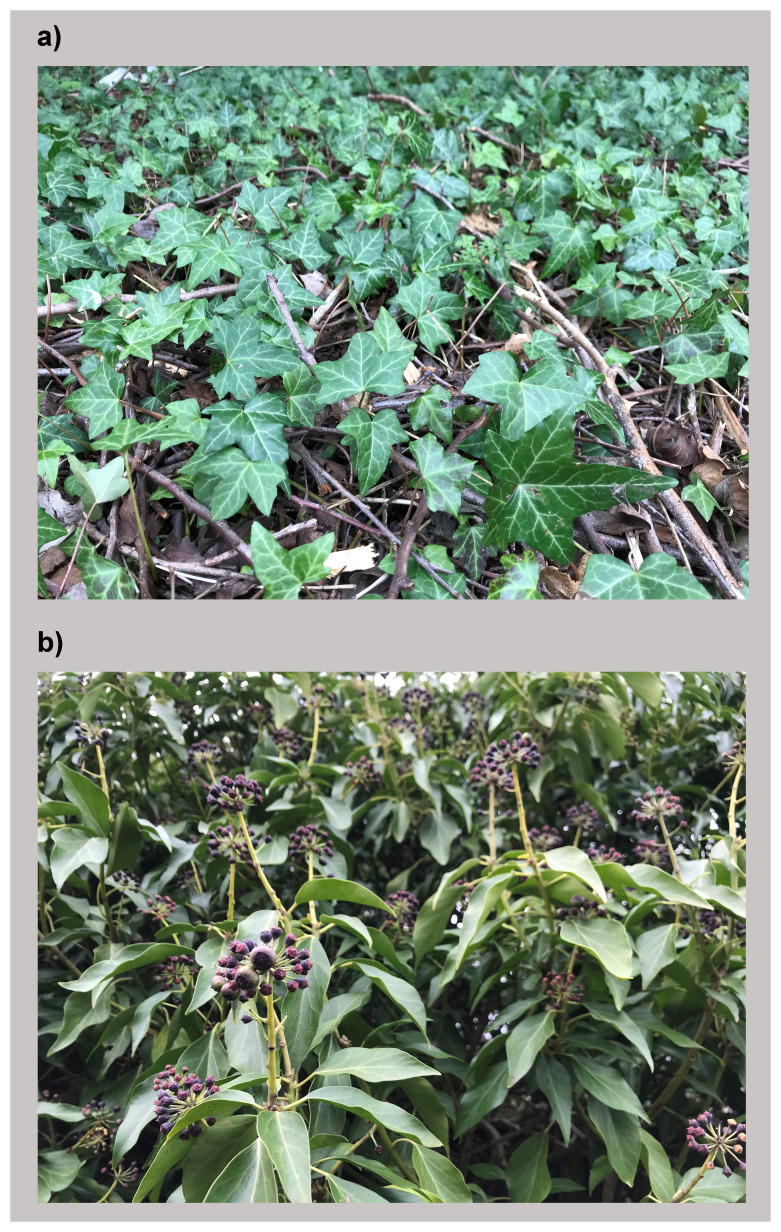
Example images of the common ivy *Hedera helix* (not the sampled specimen) growing in secondary woodland in Edinburgh. **a**) juvenile foliage. **b**) mature foliage and fruits. Photos taken by Alex Twyford.

The genome was sequenced from one *Hedera helix* specimen ( Figure 1) collected from Petersham Common, Richmond, Surrey, UK (latitude 51.45, longitude –0.30). Using flow cytometry, the genome size (1C-value) was estimated to be 1.59 pg, equivalent to 1,550 Mb. A total of 29-fold coverage in Pacific Biosciences single-molecule HiFi long reads and 65-fold coverage in 10X Genomics read clouds were generated. Primary assembly contigs were scaffolded with chromosome conformation Hi-C data. Manual assembly curation corrected 276 missing joins or mis-joins and removed 15 haplotypic duplications, reducing the assembly length by 0.63% and the scaffold number by 79.25%, and increasing the scaffold N50 by 10.54%.

**Figure 2.**
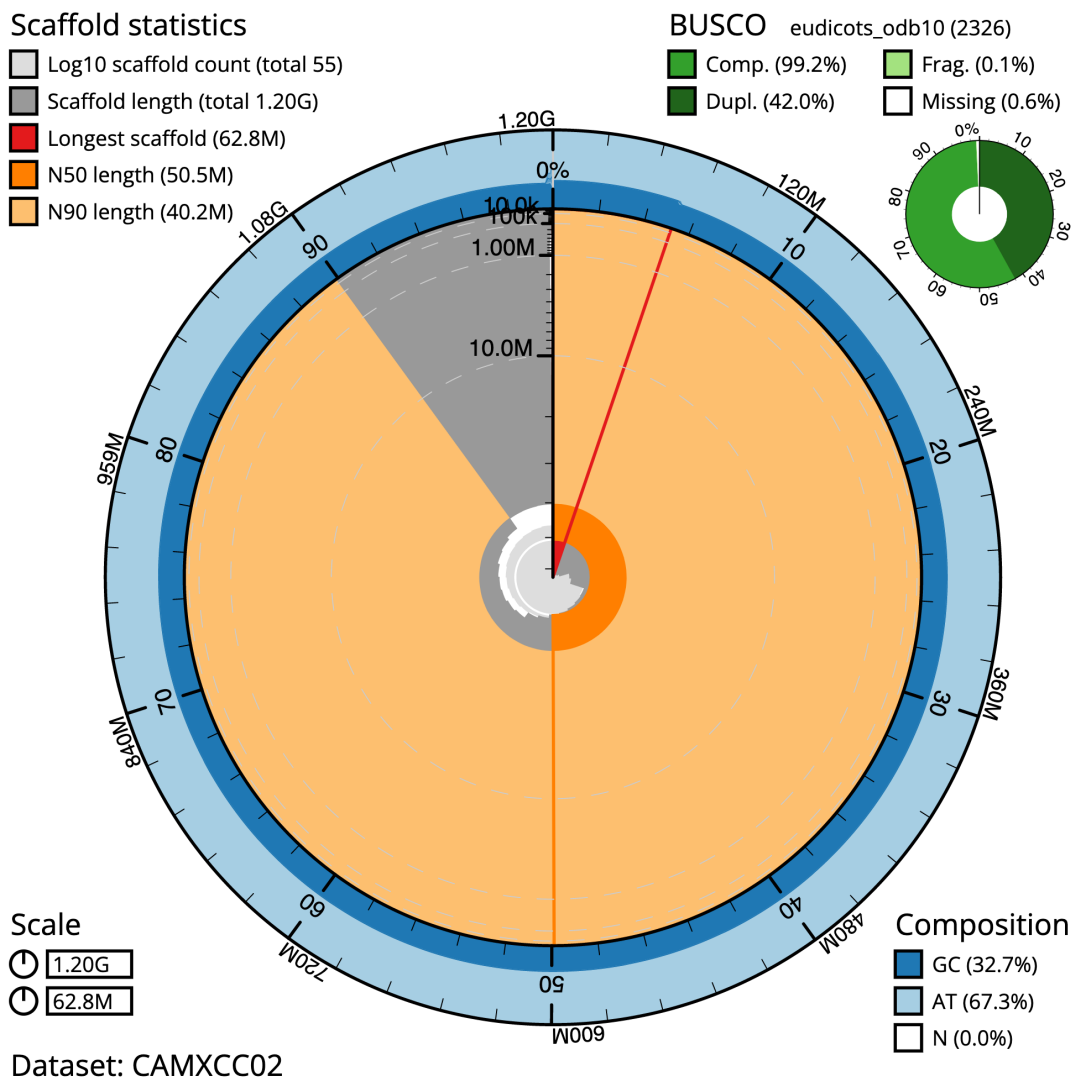
Genome assembly of *Hedera helix*, drHedHeli1.2: metrics. The BlobToolKit Snailplot shows N50 metrics and BUSCO gene completeness. The main plot is divided into 1,000 size-ordered bins around the circumference with each bin representing 0.1% of the 1,199,358,263 bp assembly. The distribution of scaffold lengths is shown in dark grey with the plot radius scaled to the longest scaffold present in the assembly (62,841,030 bp, shown in red). Orange and pale-orange arcs show the N50 and N90 scaffold lengths (50,454,882 and 40,242,199 bp), respectively. The pale grey spiral shows the cumulative scaffold count on a log scale with white scale lines showing successive orders of magnitude. The blue and pale-blue area around the outside of the plot shows the distribution of GC, AT and N percentages in the same bins as the inner plot. A summary of complete, fragmented, duplicated and missing BUSCO genes in the eudicots_odb10 set is shown in the top right. An interactive version of this figure is available at https://blobtoolkit.genomehubs.org/view/drHedHeli1.2/dataset/CAMXCC02/snail.

The final assembly has a total length of 1,199.4 Mb in 55 sequence scaffolds with a scaffold N50 of 50.5 Mb ( Table 1). Most (99.84%) of the assembly sequence was assigned to 24 chromosomal-level scaffolds. Chromosome-scale scaffolds confirmed by the Hi-C data are named in order of size ( Figure 2– Figure 5; Table 2). There is a region of low confidence on Chromosome 1 at 15–26.1 Mb consisting of several repetitive scaffolds with uncertain orientation and order. The Hi-C data indicate there is a nested heterozygous inversion on Chromosome 3 covering the approximate region 19.7–35.7 Mb. On Chromosome 20, there is a heterozygous inversion between approximately 1.6–4.4 Mb. The mitochondrial and chloroplast genomes were also assembled.

**Table 1.** Genome data for *Hedera helix*, drHedHeli1.2.

Project accession data		
Assembly identifier	drHedHeli1.2	
Species	*Hedera helix*	
Specimen	drHedHeli1	
NCBI taxonomy ID	4052	
BioProject	PRJEB47314	
BioSample ID	SAMEA7522625	
Isolate information	leaf tissue, drHedHeli1 (DNA sequencing and Hi-C) leaf tissue, drHedHeli8 (RNA-Seq)	
Assembly metrics *		*Benchmark*
Consensus quality (QV)	54.2	*≥ 50*
*k*-mer completeness	99.99%	*≥ 95%*
BUSCO **	C:99.2%[S:57.2%,D:42.0%], F:0.1%,M:0.6%,n:2,326	*C ≥ 95%*
Percentage of assembly mapped to chromosomes	99.84%	*≥ 95%*
Sex chromosomes	Not applicable.	*localised homologous pairs*
Organelles	Mitochondrial and plastid genomes assembled.	*complete single alleles*
Raw data accessions		
PacificBiosciences SEQUEL II	ERR6907987, ERR6939262	
10X Genomics Illumina	ERR6688692–ERR6688695	
Hi-C Illumina	ERR6688700	
PolyA RNA-Seq Illumina	ERR9435019	
Genome assembly		
Assembly accession	GCA_947179155.2	
*Accession of alternate haplotype*	GCA_947179205.2	
Span (Mb)	1,198.6	
Number of contigs	375	
Contig N50 length (Mb)	13.3	
Number of scaffolds	53	
Scaffold N50 length (Mb)	50.5	
Longest scaffold (Mb)	62.8	

* Assembly metric benchmarks are adapted from column VGP-2020 of “Table 1: Proposed standards and metrics for defining genome assembly quality” from ( Rhie *et al.*, 2021). ** BUSCO scores based on the eudicots_odb10 BUSCO set using v5.3.2. C = complete [S = single copy, D = duplicated], F = fragmented, M = missing, n = number of orthologues in comparison. A full set of BUSCO scores is available at https://blobtoolkit.genomehubs.org/view/drHedHeli1.2/dataset/CAMXCC02/busco.

**Figure 3.**
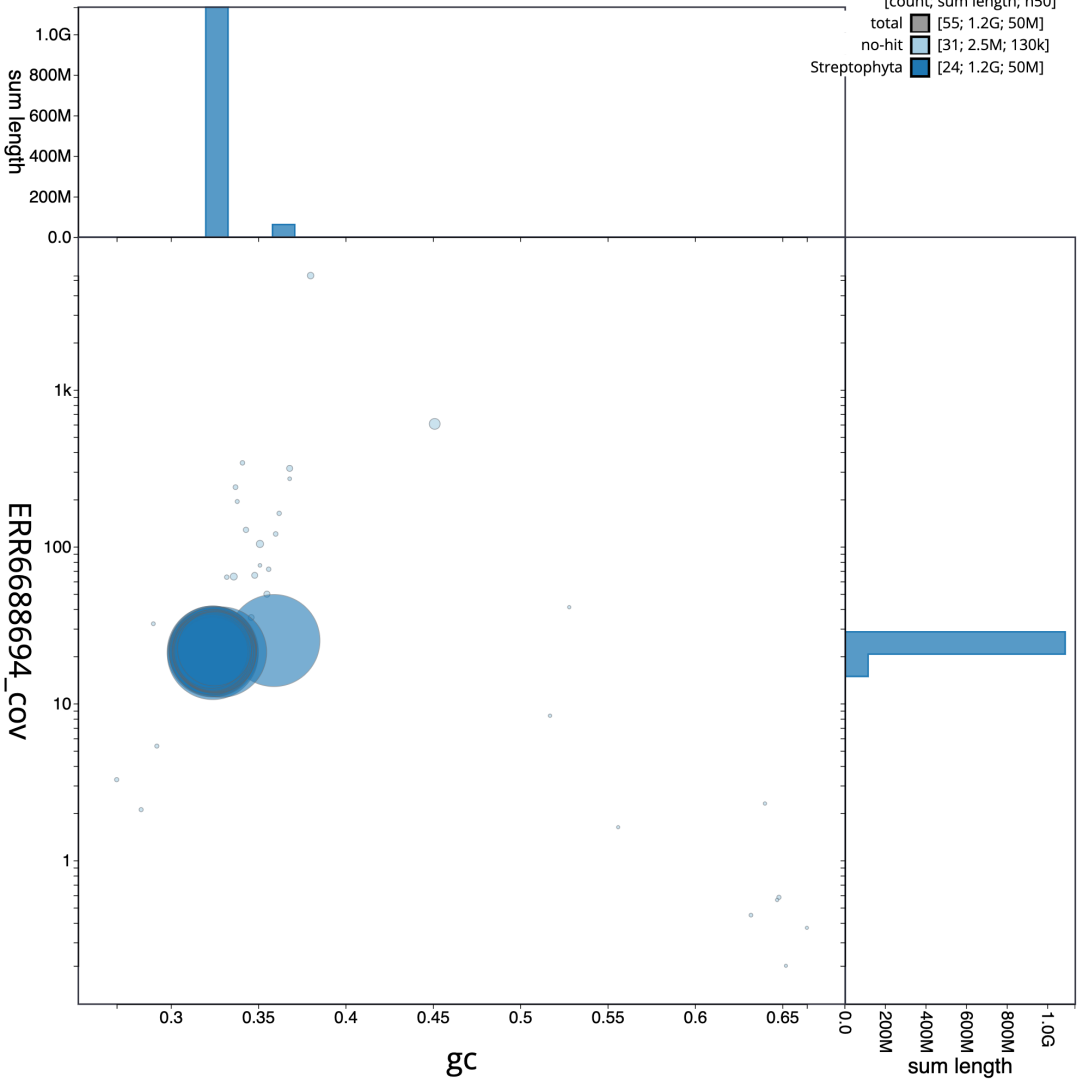
Genome assembly of *Hedera helix*, drHedHeli1.2: GC coverage. BlobToolKit GC-coverage plot. Scaffolds are coloured by phylum. Circles are sized in proportion to scaffold length. Histograms show the distribution of scaffold length sum along each axis. An interactive version of this figure is available at https://blobtoolkit.genomehubs.org/view/drHedHeli1.2/dataset/CAMXCC02/blob.

**Figure 4.**
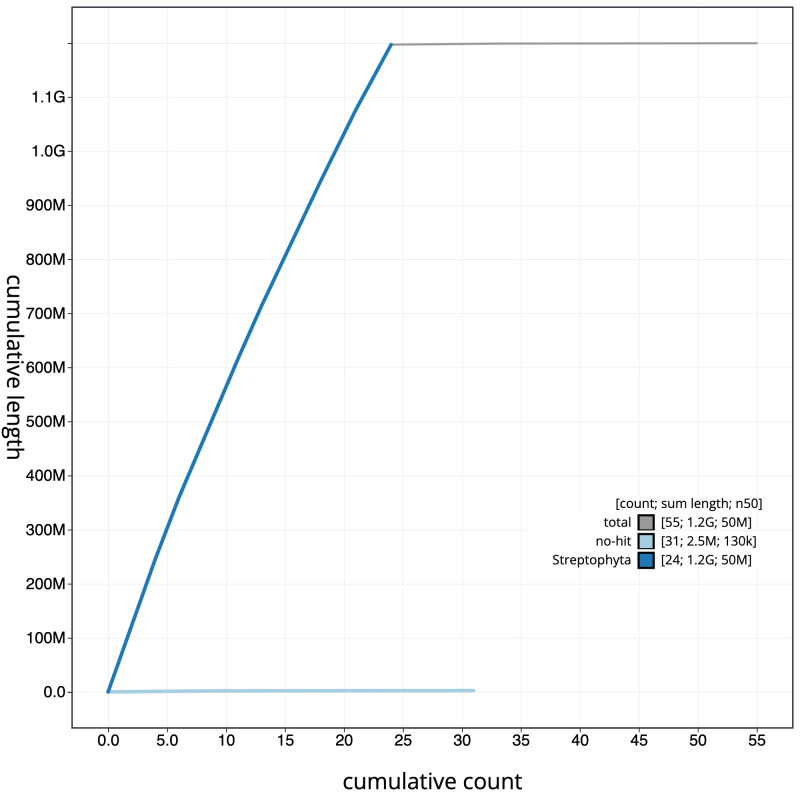
Genome assembly of *Hedera helix*, drHedHeli1.2: cumulative sequence. BlobToolKit cumulative sequence plot. The grey line shows cumulative length for all scaffolds. Coloured lines show cumulative lengths of scaffolds assigned to each phylum using the buscogenes taxrule. An interactive version of this figure is available at https://blobtoolkit.genomehubs.org/view/drHedHeli1.2/dataset/CAMXCC02/cumulative.

**Figure 5.**
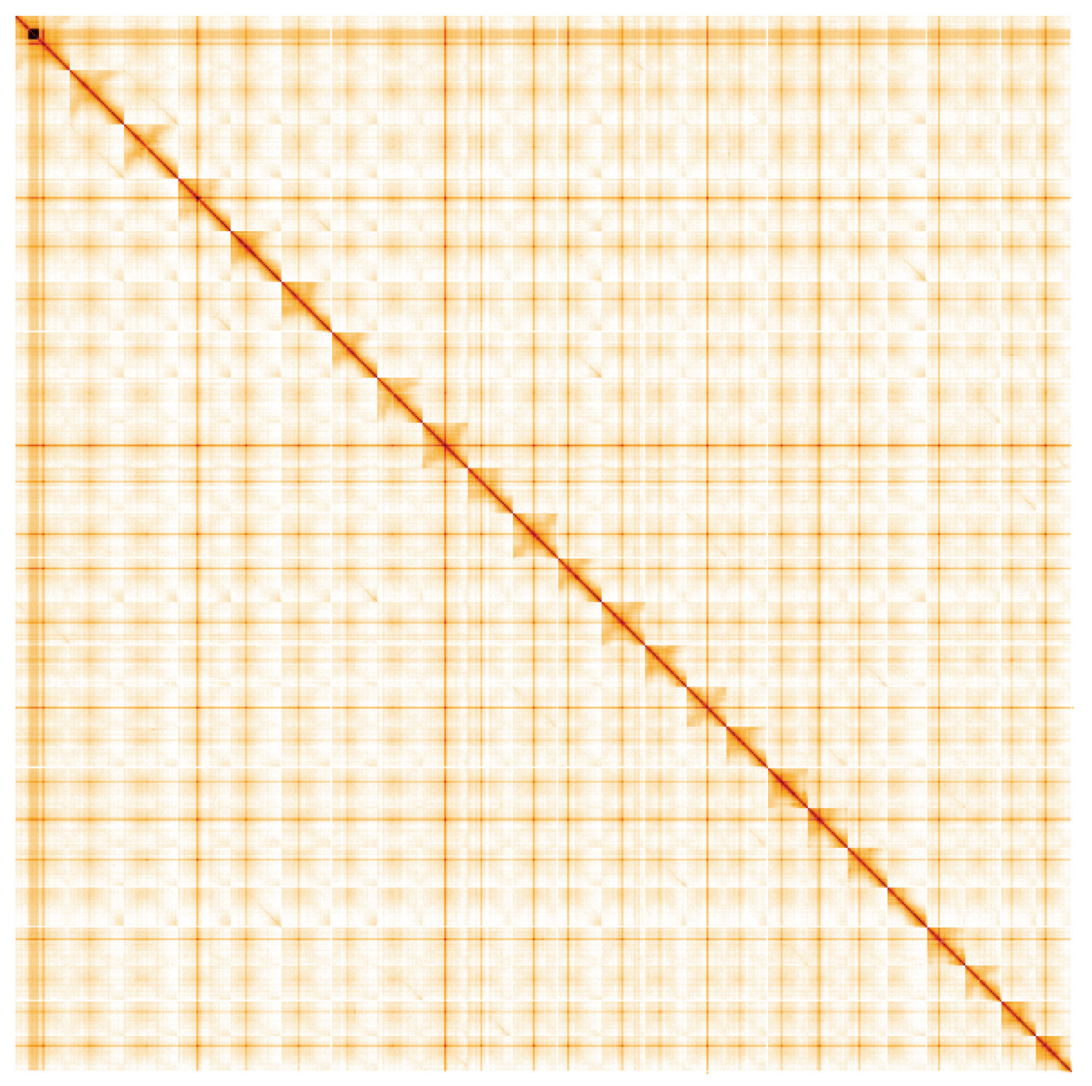
Genome assembly of *Hedera helix*, drHedHeli1.2: Hi-C contact map. Hi-C contact map of the drHedHeli1.2 assembly, visualised using HiGlass. Chromosomes are shown in order of size from left to right and top to bottom. An interactive version of this figure may be viewed at https://genome-note-higlass.tol.sanger.ac.uk/l/?d=RqLbs2mdRkulj0bOy5MAQw.

**Table 2.** Chromosomal pseudomolecules in the genome assembly of *Hedera helix*, drHedHeli1.

INSDC accession	Chromosome	Size (Mb)	GC%
OX359264.1	1	62.84	35.9
OX359265.1	2	61.43	32.4
OX359266.1	3	61.1	32.4
OX359267.1	4	60.35	32.9
OX359268.1	5	57.05	32.4
OX359269.1	6	56.44	32.4
OX359270.1	7	51.84	32.4
OX359271.1	8	51.67	32.4
OX359272.1	9	51.29	32.5
OX359273.1	10	50.79	32.3
OX359274.1	11	50.45	32.5
OX359275.1	12	49.42	32.5
OX359276.1	13	48.73	32.4
OX359277.1	14	46.9	32.5
OX359278.1	15	46.45	32.6
OX359279.1	16	45.93	32.4
OX359280.1	17	45.65	32.4
OX359281.1	18	45.28	32.5
OX359282.1	19	44.67	32.5
OX359283.1	20	44.38	32.4
OX359284.1	21	43.64	32.3
OX359285.1	22	40.24	32.4
OX359286.1	23	40.2	32.3
OX359287.1	24	40.14	32.5
OX381723.1	mitochondrion	0.61	45.1
OX381724.1	plastid	0.16	38
-	unplaced	1.7	36.8

The assembly has a BUSCO v5.3.2 ( Manni *et al.*, 2021) completeness of 99.2% (single 57.2%, duplicated 42.0%) using the eudicots_odb10 reference set. While not fully phased, the assembly deposited is of one haplotype. Contigs corresponding to the second haplotype have also been deposited.

The estimated Quality Value (QV) of the final assembly is 54.2 with *k*-mer completeness of 99.99%, and the assembly has a BUSCO v5.3.2 completeness of 99.2% (single = 57.2%, duplicated = 42.0%), using the eudicots_odb10 reference set ( *n* = 2,326).

Metadata for specimens, spectral estimates, sequencing runs, contaminants and pre-curation assembly statistics can be found at https://links.tol.sanger.ac.uk/species/4052.

## Methods

### Sample acquisition, genome size estimation and nucleic acid extraction

A *Hedera helix specimen* (drHedHeli1) was collected from Petersham Common, Richmond, Surrey, UK (latitude 51.45, longitude –0.30) on 8 September 2020. The specimen was picked by hand from a beech woodland habitat by Maarten Christenhusz (Royal Botanic Gardens, Kew) collection number 9099. The specimen was identified by Maarten Christenhusz based on its morphology and preserved by freezing at –80°C.

A second specimen (drHedHeli8) was collected from the Royal Botanic Garden Edinburgh (Inverleith) (latitude 55.96, longitude –3.20) on 23 November 2020 by David Bell (Royal Botanic Garden Edinburgh). The specimen was identified by David Bell based on its morphology and flash-frozen in liquid nitrogen. This specimen was used for RNA sequencing.

The genome size was estimated by flow cytometry using the fluorochrome propidium iodide and following the ‘one-step’ method outlined in Pellicer *et al.* (2021). Specifically for this species, the General Purpose Buffer (GPB) supplemented with 3% PVP and 0.08% (v/v) beta-mercaptoethanol was used for isolation of nuclei ( Loureiro *et al.*, 2007), and the internal calibration standard was *Petroselinum crispum* ‘Champion Moss Curled’ with an assumed 1C-value of 2,200 Mb ( Obermayer *et al.*, 2002).

DNA was extracted at the Tree of Life laboratory, Wellcome Sanger Institute (WSI). The drHedHeli1 sample was weighed and dissected on dry ice with tissue set aside for Hi-C sequencing. Leaf tissue was cryogenically disrupted to a fine powder using a Covaris cryoPREP Automated Dry Pulveriser, receiving multiple impacts. High molecular weight (HMW) DNA was extracted using the Qiagen Plant MagAttract HMW DNA extraction kit. Low molecular weight DNA was removed from a 20 ng aliquot of extracted DNA using the 0.8X AMpure XP purification kit prior to 10X Chromium sequencing; a minimum of 50 ng DNA was submitted for 10X sequencing. HMW DNA was sheared into an average fragment size of 12–20 kb in a Megaruptor 3 system with speed setting 30. Sheared DNA was purified by solid-phase reversible immobilisation using AMPure PB beads with a 1.8× ratio of beads to sample to remove the shorter fragments and concentrate the DNA sample. The concentration of the sheared and purified DNA was assessed using a Nanodrop spectrophotometer and Qubit Fluorometer and Qubit dsDNA High Sensitivity Assay kit. Fragment size distribution was evaluated by running the sample on the FemtoPulse system.

RNA was extracted from leaf tissue of drHedHeli8 in the Tree of Life Laboratory at the WSI using TRIzol, according to the manufacturer’s instructions. RNA was then eluted in 50 μl RNAse-free water and its concentration assessed using a Nanodrop spectrophotometer and Qubit Fluorometer using the Qubit RNA Broad-Range (BR) Assay kit. Analysis of the integrity of the RNA was done using Agilent RNA 6000 Pico Kit and Eukaryotic Total RNA assay.

### Sequencing

Pacific Biosciences HiFi circular consensus and 10X Genomics read cloud DNA sequencing libraries were constructed according to the manufacturers’ instructions. Poly(A) RNA-Seq libraries were constructed using the NEB Ultra II RNA Library Prep kit. DNA and RNA sequencing was performed by the Scientific Operations core at the WSI on Pacific Biosciences SEQUEL II (HiFi), Illumina HiSeq 4000 (RNA-Seq) and Illumina NovaSeq 6000 (10X) instruments. Hi-C data were also generated from drHedHeli1 using the Arima v2 kit and sequenced on the Illumina NovaSeq 6000 instrument.

### Genome assembly, curation and evaluation

Assembly was carried out with Hifiasm ( Cheng *et al.*, 2021) and haplotypic duplication was identified and removed with purge_dups ( Guan *et al.*, 2020). One round of polishing was performed by aligning 10X Genomics read data to the assembly with Long Ranger ALIGN, calling variants with FreeBayes ( Garrison & Marth, 2012). The assembly was then scaffolded with Hi-C data ( Rao *et al.*, 2014) using SALSA2 ( Ghurye *et al.*, 2019). The assembly was checked for contamination and corrected using the gEVAL system ( Chow *et al.*, 2016) as described previously ( Howe *et al.*, 2021). Manual curation was performed using gEVAL, HiGlass ( Kerpedjiev *et al.*, 2018) and Pretext ( Harry, 2022). The mitochondrial and chloroplast genomes were assembled using MBG ( Rautiainen & Marschall, 2021) from PacBio HiFi reads mapping to related genomes. A representative circular sequence was selected for each from the graph based on read coverage.

A Hi-C map for the final assembly was produced using bwa-mem2 ( Vasimuddin *et al.*, 2019) in the Cooler file format ( Abdennur & Mirny, 2020). To assess the assembly metrics, the k-mer completeness and QV consensus quality values were calculated in Merqury ( Rhie *et al.*, 2020). This work was done using Nextflow ( Di Tommaso *et al.*, 2017) DSL2 pipelines “sanger-tol/readmapping” ( Surana *et al.*, 2023a) and “sanger-tol/genomenote” ( Surana *et al.*, 2023b). The genome was analysed within the BlobToolKit environment ( Challis *et al.*, 2020) and BUSCO scores ( Manni *et al.*, 2021; Simão *et al.*, 2015) were calculated.

Table 3 contains a list of relevant software tool versions and sources.

**Table 3.** Software tools: versions and sources.

Software tool	Version	Source
BlobToolKit	4.0.7	https://github.com/blobtoolkit/blobtoolkit
BUSCO	5.3.2	https://gitlab.com/ezlab/busco
FreeBayes	1.3.1-17-gaa2ace8	https://github.com/freebayes/freebayes
gEVAL	N/A	https://geval.org.uk/
Hifiasm	0.15.3-r339	https://github.com/chhylp123/hifiasm
HiGlass	1.11.6	https://github.com/higlass/higlass
Long Ranger ALIGN	2.2.2	https://support.10xgenomics.com/genome-exome/ software/pipelines/latest/advanced/other-pipelines
Merqury	MerquryFK	https://github.com/thegenemyers/MERQURY.FK
MBG	-	https://github.com/maickrau/MBG
PretextView	0.2	https://github.com/wtsi-hpag/PretextView
purge_dups	1.2.3	https://github.com/dfguan/purge_dups
SALSA	2.2	https://github.com/salsa-rs/salsa

### Wellcome Sanger Institute – Legal and Governance

The materials that have contributed to this genome note have been supplied by a Darwin Tree of Life Partner. The submission of materials by a Darwin Tree of Life Partner is subject to the **‘Darwin Tree of Life Project Sampling Code of Practice’**, which can be found in full on the Darwin Tree of Life website here. By agreeing with and signing up to the Sampling Code of Practice, the Darwin Tree of Life Partner agrees they will meet the legal and ethical requirements and standards set out within this document in respect of all samples acquired for, and supplied to, the Darwin Tree of Life Project. 

Further, the Wellcome Sanger Institute employs a process whereby due diligence is carried out proportionate to the nature of the materials themselves, and the circumstances under which they have been/are to be collected and provided for use. The purpose of this is to address and mitigate any potential legal and/or ethical implications of receipt and use of the materials as part of the research project, and to ensure that in doing so we align with best practice wherever possible. The overarching areas of consideration are: 

•   Ethical review of provenance and sourcing of the material

•   Legality of collection, transfer and use (national and international)

Each transfer of samples is further undertaken according to a Research Collaboration Agreement or Material Transfer Agreement entered into by the Darwin Tree of Life Partner, Genome Research Limited (operating as the Wellcome Sanger Institute), and in some circumstances other Darwin Tree of Life collaborators.

## Data Availability

European Nucleotide Archive: *Hedera helix*. Accession number PRJEB47314; https://identifiers.org/ena.embl/PRJEB47314. ( Wellcome Sanger Institute, 2022) The genome sequence is released openly for reuse. The *Hedera helix* genome sequencing initiative is part of the Darwin Tree of Life (DToL) project. All raw sequence data and the assembly have been deposited in INSDC databases. The genome will be annotated using available RNA-Seq data and presented through the Ensembl pipeline at the European Bioinformatics Institute. Raw data and assembly accession identifiers are reported in Table 1.

## Author information

Members of the Royal Botanic Gardens Kew Genome Acquisition Lab are listed here: https://doi.org/10.5281/zenodo.4786680.

Members of the Royal Botanic Garden Edinburgh Genome Acquisition Lab are listed here: https://doi.org/10.5281/zenodo.4786682.

Members of the Plant Genome Sizing collective are listed here: https://doi.org/10.5281/zenodo.7994306.

Members of the Darwin Tree of Life Barcoding collective are listed here: https://doi.org/10.5281/zenodo.4893703.

Members of the Wellcome Sanger Institute Tree of Life programme are listed here: https://doi.org/10.5281/zenodo.4783585.

Members of Wellcome Sanger Institute Scientific Operations: DNA Pipelines collective are listed here: https://doi.org/10.5281/zenodo.4790455.

Members of the Tree of Life Core Informatics collective are listed here: https://doi.org/10.5281/zenodo.5013541.

## References

[ref-23] Abdennur N Mirny LA : Cooler: Scalable storage for Hi-C data and other genomically labeled arrays. *Bioinformatics.* 2020;36(1):311–316. 10.1093/bioinformatics/btz540 31290943 PMC8205516

[ref-1] Biggerstaff MS Beck CW : Effects of English Ivy ( *Hedera helix*) on Seed Bank Formation and Germination. *Am Midl Nat.* 2007;157(2):250–257. 10.1674/0003-0031(2007)157[250:EOEIHH]2.0.CO;2

[ref-24] Challis R Richards E Rajan J : BlobToolKit – interactive quality assessment of genome assemblies. G3: Genes, Genomes. Genetics,2020;10(4):1361–1374. 10.1534/g3.119.400908 32071071 PMC7144090

[ref-2] Cheng H Concepcion GT Feng X : Haplotype-resolved *de novo* assembly using phased assembly graphs with hifiasm. *Nat Methods.* 2021;18(2):170–175. 10.1038/s41592-020-01056-5 33526886 PMC7961889

[ref-3] Chow W Brugger K Caccamo M : gEVAL—a web-based browser for evaluating genome assemblies. *Bioinformatics.* 2016;32(16):2508–2510. 10.1093/bioinformatics/btw159 27153597 PMC4978925

[ref-25] Di Tommaso P Chatzou M Floden EW : Nextflow enables reproducible computational workflows. *Nat Biotechnol.* 2017;35(4):316–319. 10.1038/nbt.3820 28398311

[ref-4] Garrison E Marth G : Haplotype-based variant detection from short-read sequencing.2012. 10.48550/arXiv.1207.3907

[ref-5] Ghurye J Rhie A Walenz BP : Integrating Hi-C links with assembly graphs for chromosome-scale assembly. *PLoS Comput Biol.* 2019;15(8): e1007273. 10.1371/journal.pcbi.1007273 31433799 PMC6719893

[ref-6] Guan D McCarthy SA Wood J : Identifying and removing haplotypic duplication in primary genome assemblies. *Bioinformatics.* 2020;36(9):2896–2898. 10.1093/bioinformatics/btaa025 31971576 PMC7203741

[ref-7] Harry E : PretextView (Paired REad TEXTure Viewer): A desktop application for viewing pretext contact maps.2022; [Accessed 19 October 2022]. Reference Source

[ref-8] Howe K Chow W Collins J : Significantly improving the quality of genome assemblies through curation. *GigaScience.* Oxford University Press,2021;10(1): giaa153. 10.1093/gigascience/giaa153 33420778 PMC7794651

[ref-9] Kerpedjiev P Abdennur N Lekschas F : HiGlass: web-based visual exploration and analysis of genome interaction maps. *Genome Biol.* 2018;19(1): 125. 10.1186/s13059-018-1486-1 30143029 PMC6109259

[ref-10] Loureiro J Rodriguez E Dolezel J : Two new nuclear isolation buffers for plant DNA flow cytometry: A test with 37 species. *Ann Bot.* 2007;100(4):875–888. 10.1093/aob/mcm152 17684025 PMC2749623

[ref-11] Manni M Berkeley MR Seppey M : BUSCO update: Novel and streamlined workflows along with broader and deeper phylogenetic coverage for scoring of eukaryotic, prokaryotic, and viral genomes. *Mol Biol Evol.* 2021;38(10):4647–4654. 10.1093/molbev/msab199 34320186 PMC8476166

[ref-12] Metcalfe DJ : *Hedera helix* L. *J Ecol.* 2005;93(3):632–648. 10.1111/j.1365-2745.2005.01021.x

[ref-13] Obermayer R Leitch IJ Hanson L : Nuclear DNA C-values in 30 species double the familial representation in pteridophytes. *Ann Bot.* 2002;90(2):209–217. 10.1093/aob/mcf167 12197518 PMC4240412

[ref-14] Pellicer J Powell RF Leitch IJ : The application of flow cytometry for estimating genome Size, Ploidy Level Endopolyploidy, and Reproductive Modes in Plants.In: Besse, P. (ed.) *Methods in Molecular Biology (Clifton, N.J.)*. New York, NY: Humana,2021;2222:325–361. 10.1007/978-1-0716-0997-2_17 33301101

[ref-15] Rao SSP Huntley MH Durand NC : A 3D map of the human genome at kilobase resolution reveals principles of chromatin looping. *Cell.* 2014;159(7):1665–1680. 10.1016/j.cell.2014.11.021 25497547 PMC5635824

[ref-16] Rautiainen M Marschall T : MBG: Minimizer-based sparse de Bruijn Graph construction. *Bioinformatics.* 2021;37(16):2476–2478. 10.1093/bioinformatics/btab004 33475133 PMC8521641

[ref-26] Rhie A Walenz BP Koren S : Merqury: Reference-free quality, completeness, and phasing assessment for genome assemblies. *Genome Biol.* 2020;21(1):245. 10.1186/s13059-020-02134-9 32928274 PMC7488777

[ref-17] Rhie A McCarthy SA Fedrigo O : Towards complete and error-free genome assemblies of all vertebrate species. *Nature.* 2021;592(7856):737–746. 10.1038/s41586-021-03451-0 33911273 PMC8081667

[ref-18] Schäffner KH Nagl W : Differential DNA replication involved in transition from juvenile to adult phase in *Hedera helix* ( *Araliaceae*).In: *Plant Syst Evol*.1979;2:105–110. 10.1007/978-3-7091-8556-8_8

[ref-27] Simão FA Waterhouse RM Ioannidis P : BUSCO: assessing genome assembly and annotation completeness with single-copy orthologs. *Bioinformatics.* 2015;31(19):3210–3212. 10.1093/bioinformatics/btv351 26059717

[ref-28] Surana P Muffato M Qi G : sanger-tol/readmapping: sanger-tol/readmapping v1.1.0 - Hebridean Black (1.1.0). *Zenodo.* 2023a; [Accessed 21 July 2023]. 10.5281/zenodo.7755665

[ref-29] Surana P Muffato M Sadasivan Baby C : sanger-tol/genomenote (v1.0.dev). *Zenodo.* 2023b; [Accessed 21 July 2023]. 10.5281/zenodo.6785935

[ref-19] Stace C : New flora of the British Isles.Cambridge: Cambridge University Press.2010. Reference Source

[ref-20] Sun H Li F Xu Z : *De novo* leaf and root transcriptome analysis to identify putative genes involved in triterpenoid saponins biosynthesis in *Hedera helix* L. *PLoS One.* 2017;12(8): e0182243. 10.1371/journal.pone.0182243 28771546 PMC5542655

[ref-21] Twyford AD : Parasitic plants. *Curr Biol.* 2018;28(16):R857–R859. 10.1016/j.cub.2018.06.030 30130499

[ref-30] Vasimuddin Md Misra S Li H : Efficient Architecture-Aware Acceleration of BWA-MEM for Multicore Systems. In: *2019 IEEE International Parallel and Distributed Processing Symposium (IPDPS).* * IEEE,* 2019;314–324. 10.1109/IPDPS.2019.00041

[ref-22] Wellcome Sanger Institute: The genome sequence of common ivy, *Hedera helix* (Linnaeus 1753). * European Nucleotide Archive*, [dataset], accession number PRJEB47314.2022.

