# Supplementary material for: The genome sequence of common ivy, Hedera helix L., 1753
Source: Wellcome Open Res. 2023 Jul 26;8:325. [Version 1] doi: 10.12688/wellcomeopenres.19662.1 (PMC10690035; doi:10.12688/wellcomeopenres.19662.1)
Supplement: Peer review history

# Reviewer report: Reviewer response for version 1

**DOI:** 10.21956/wellcomeopenres.21779.r68208

**Published:** 2023-11-30

**Authors:** Shu-Miaw Chaw

**Affiliations:** 1 Biodiversity Research Center Academia Sinica, Taipei City, Taipei City, Taiwan

This manuscript reports the first reference genome sequence of Hedera helix L. (English ivy or common ivy). As English ivy and other introduced ivy species (have escaped from gardens) are now introduced (or probably invasive) aliens globally, and the taxonomy of ivy is complex. Completion and annotation of a high-quality genome sequence of the common ivy will certainly be an important resource to understand not only the development of several morphological characters (such as leaf lobe and climbing habit), but also genes involved in invasiveness, biosynthesis of secondary metabolites as well as interaction mechanism with its parasitic plants. To this end, this manuscript is well written, concise, and clear. It will be appealing to general readers. The methodology about assembly and its quality verification are correctly described with detailed explanation and logics. The figures and tables are excellently illustrated with high resolution and quality, though it is hard for me to understand Figure 3. I strongly recommend acceptance of this manuscript for indexing.

Are sufficient details of methods and materials provided to allow replication by others?

Yes

Is the rationale for creating the dataset(s) clearly described?

Yes

Are the datasets clearly presented in a useable and accessible format?

Yes

Are the protocols appropriate and is the work technically sound?

Yes

Reviewer Expertise:

Plant comparative genomics

I confirm that I have read this submission and believe that I have an appropriate level of expertise to confirm that it is of an acceptable scientific standard.

# Reviewer report: Reviewer response for version 1

**DOI:** 10.21956/wellcomeopenres.21779.r70216

**Published:** 2023-11-24

**Authors:** Yoshinori Fukasawa

**Affiliations:** 1 Center for Center for Bioscience Research and Education, Utsunomiya University, Tochigi, Japan

The authors present the genome assembly of common ivy, *Hedera helix* (hereafter *H. helix*), because it provides a comprehensive understanding of the genetic makeup of this species. The assembly is nearly 1.2 Gb in size and is scaffolded into 24 chromosomal pseudomolecules (55 sequence scaffolds in total) using multiple platforms. In addition, the mitochondrial and plastid genomes have also been assembled. This complete genome assembly could be used as a fundamental resource in studies of the common ivy.

 From a technical perspective, the genome assembly process for *H. helix* was performed using a combination of long-read sequencing technologies, Hi-C scaffolding, and manual curation. The authors used Hifiasm for the initial assembly, which was then polished using 10X Genomics read data and FreeBayes variant calling. The assembly was then scaffolded using Hi-C data, and finally manual curation was performed using gEVAL, HiGlass, and Pretext.

 One of the major challenges the researchers faced was the complexity of the genome such as the presence of haplotypic duplications, and the need for manual curation to correct errors in the assembly. Standard contiguity metrics such as N50 and BUSCO show convincing values.

 Minor comments:

1. Polishing the HiFi assembly with short read data poses a risk of introducing technical errors, especially at this level of accuracy. To address this issue, a recent study utilized a hybrid variant calling model that can handle both PacBio HiFi and Illumina short reads simultaneously (Mc Cartney *et al.*, 2022 ^1^). It would be beneficial for the authors to provide a citation to support this approach or show the improvement from polishing in the assembly.
2. Could you please specify which read set(s) were used for the k-mer computation in the QV calculation with Merqury? This detail seems to be missing.
3. Could you clarify if the pseudomolecules extend from telomere to telomere, at least for some of them? This would be helpful to know.

Are sufficient details of methods and materials provided to allow replication by others?

Yes

Is the rationale for creating the dataset(s) clearly described?

Yes

Are the datasets clearly presented in a useable and accessible format?

Yes

Are the protocols appropriate and is the work technically sound?

Yes

Reviewer Expertise:

genomics, bioinformatics

I confirm that I have read this submission and believe that I have an appropriate level of expertise to confirm that it is of an acceptable scientific standard.

## References

[1] : Chasing perfection: validation and polishing strategies for telomere-to-telomere genome assemblies. *Nat Methods* .2022;19(6) : 10.1038/s41592-022-01440-3 687-695 10.1038/s41592-022-01440-3 35361931PMC9812399
